# The Potential of Fibroblast Activation Protein-Targeted Imaging as a Biomarker of Cardiac Remodeling and Injury

**DOI:** 10.1007/s11886-023-01869-8

**Published:** 2023-05-01

**Authors:** Maday Fernandez Mayola, James T. Thackeray

**Affiliations:** Department of Nuclear Medicine, Hannover Medical School, Translational Cardiovascular Molecular Imaging, Carl Neuberg Str 1, 30625 Hannover, Germany

**Keywords:** Cardiac fibroblast, Heart failure, Molecular imaging, Myocardial infarction, Fibroblast activation protein, Fibrosis

## Abstract

**Purpose of Review:**

Cardiovascular disease features adverse fibrotic processes within the myocardium, leading to contractile dysfunction. Activated cardiac fibroblasts play a pivotal role in the remodeling and progression of heart failure, but conventional diagnostics struggle to identify early changes in cardiac fibroblast dynamics. Emerging imaging methods visualize fibroblast activation protein (FAP) as a marker of activated fibroblasts, enabling non-invasive quantitative measurement of early cardiac remodeling.

**Recent Findings:**

Retrospective analysis of oncology patient cohorts has identified cardiac uptake of FAP radioligands in response to various cardiovascular conditions. Small scale studies in dedicated cardiac populations have revealed FAP upregulation in injured myocardium, wherein the area of upregulation predicts subsequent ventricle dysfunction. Recent studies have demonstrated that silencing of FAP-expressing fibroblasts can reverse cardiac fibrosis in disease models.

**Summary:**

The parallel growth of FAP-targeted imaging and therapy provides the opportunity for imaging-based monitoring and refinement of treatments targeting cardiac fibroblast activation.

## Introduction

The heart comprises a mosaic of cellular subtypes that help maintain myocyte structure and contractile function. Recent single-cell RNA sequencing studies identify 15–20% of cells in the adult heart as cardiac fibroblasts [1], which themselves constitute a range of different functional populations from quiescent to active structural remodelers. In conditions of cardiac damage, the proportion of fibroblasts changes which contribute to adverse remodeling of the left ventricle characterized by replacement fibrosis or scar formation and reactive or interstitial fibrosis [2, 3]. As such, activated cardiac fibroblasts are an attractive therapeutic target to modulate the remodeling process and improve functional outcomes [4, 5]. Recent evidence in mouse models of cardiac fibrosis suggests the capacity to selectively target activated fibroblasts to arrest or even reverse reactive fibrosis [6, 7•, 8]. But measuring cardiac fibroblast activation and response to novel targeted anti-fibrotic therapy is hindered by the lack of non-invasive markers of adverse cardiac fibroblast activity.

Imaging of cardiac fibrosis has been largely limited to magnetic resonance-based measurements of T1 relaxation and extracellular volume, which provide only crude indications of tissue composition [9]. Prolonged T1 relaxation time or elevated extracellular volume are associated with cardiac fibrosis but are also influenced by localized immune cell activity and edema [10, 11]. Accordingly, novel imaging methods in parallel with new therapeutics are desirable to provide a clearer understanding of temporal and targeted efficacy. Imaging agents targeting the serine protease fibroblast activation protein (FAP) have seen a recent explosion in application in cardiovascular disease. Here, we will discuss the current evidence supporting the use of FAP-targeted radionuclide imaging in cardiology, highlight the limitations of present research, and expound on the steps necessary to fully exploit the potential of fibroblast activation protein imaging as a biomarker of ventricle remodeling.

## Fibroblast Activation Protein

**Fig. 1:**
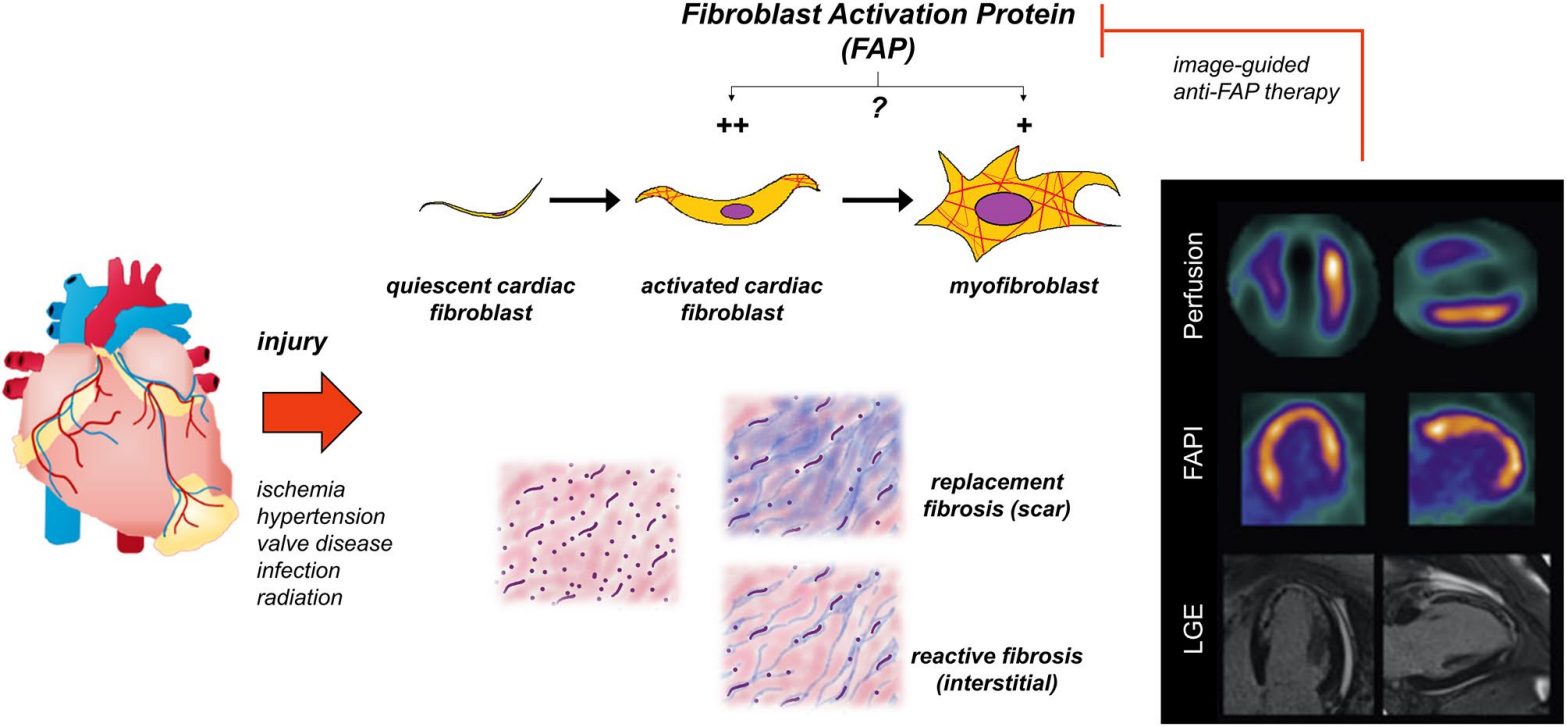
Role of fibroblast activation after myocardial injury and potential as an imaging biomarker of subsequent ventricle remodeling. After injury, quiescent cardiac fibroblasts undergo activation and proliferation. These activated fibroblasts later mature to myofibroblasts which contribute to extracellular matrix remodeling and the dual processes of replacement fibrosis or scar generation and reactive fibrosis or interstitial fibrosis. Fibroblast activation protein (FAP) is upregulated by cardiac fibroblasts after injury, but the precise fibroblast subtypes with enriched FAP expression remain equivocal. FAP imaging visualizes activated fibroblasts with an aim to monitor novel anti-fibrotic therapies and identify optimal targeting and timing of intervention

Quiescent cardiac fibroblasts respond to tissue injury undergoing phenotypic changes and differentiation to active cell types including myofibroblasts [5]. These active cells secrete extracellular matrix proteins including collagen that are essential for effective wound healing. After experimental myocardial infarction, resident cardiac fibroblasts undergo rapid proliferation and differentiation reaching maximum concentrations at 2–4 days after injury [5]. While myofibroblasts are traditionally identified by expression of α-smooth muscle actin localized to the infarct region, growing evidence implicates transitional states of activated cardiac fibroblasts that migrate to the infarct border zone and participate in wound healing. Expression of the prolyl-specific serine peptidase fibroblast activation protein (FAP) is a hallmark of activated fibroblasts as seen after myocardial infarction [12]. FAP expression is influenced by cytokines like transforming growth factor-β, also associated with migration and transdifferentiation of quiescent cardiac fibroblasts [12]. Moreover, elevated FAP-positive cardiac fibroblasts have been identified in experimental pressure overload heart failure in mice after transverse aortic constriction [8], suggesting a concurrent role in interstitial as well as replacement fibrosis. While soluble FAP may be measured in serum, tissue-level FAP expression can only be measured from tissue biopsy, limiting its value as a biomarker of progressive fibrosis (Fig. 1).

## FAP-Targeted Radiopharmaceuticals

The broad spectrum of fibrotic disease and particularly involvement in cancer rendered FAP a potential target for oncology imaging and therapy targeting tumor-associated fibroblasts. After initial assessment of radiolabeled antibodies in the mid-1990s [13], small molecule radioligands targeting FAP have seen substantial growth in the last decade, particularly quinoline-based FAP inhibitor (FAPI) series of compounds [14, 15]. These chelator-conjugated small molecule FAPI agents exhibit theragnostic potential with the combined benefit of imaging and radiotherapy delivery to FAP-expressing tumor stromal cells [14]. One of the lead compounds ^68^Ga-FAPI-04 has seen widespread application in clinical cancer [16]. Further refinement of the FAPI series has seen the development of fluorine-18 labeled FAP ligands including [^18^F]AlF-FAPI-74 [17, 18]. The armamentarium has subsequently expanded with other novel compounds that have emerged in recent years [19, 20]. The success of these agents in oncology has fueled interest in expanding clinical application into other disorders characterized by fibrosis including cardiovascular disease (Table 1).

**Table 1:** Summary of FAP-targeted imaging agents

**Compound**	**Population**	**Observations**	**Ref**
^131^I-F19 Ab (sibrotuzumab)	Colorectal carcinoma, non-small cell lung cancer	-Renal excretion -High signal to background ratio -Potential hepatic degradation	[13, 40]
^99m^Tc-FAPI-34	Ovarian cancer Pancreatic cancer	-Renal excretion -Visible tumor signal	[20]
^68^Ga-FAPI-02	Mixed cancer cohort	-Renal excretion -Minimal background -Modest tumor signal	[41]
^68^Ga-FAPI-04	Mixed cancer cohort	-Renal excretion -Low background signal -High target to background ratio -Favorable kinetics for theragnostics	[14]
^68^Ga-FAPI-46	Mixed cancer cohort	-Renal excretion -Low background signal -High target to background ratio -Favorable kinetics for theragnostics	[14, 16, 41]
^18^F-AlF-NOTA-FAPI-04	Mixed cancer cohort	-Renal and hepatobiliary excretion -Non-specific signal in submandibular glands, thyroid, and pancreas -Modest target to background ratio	[42]
^18^F-AlF-NOTA-FAPI-74	Mixed cancer cohort	-Renal excretion -Low background signal -High target to background ratio -Slower blood clearance	[18]

## Retrospective Cardiovascular Analysis of Oncology Patients

**Fig. 2:**
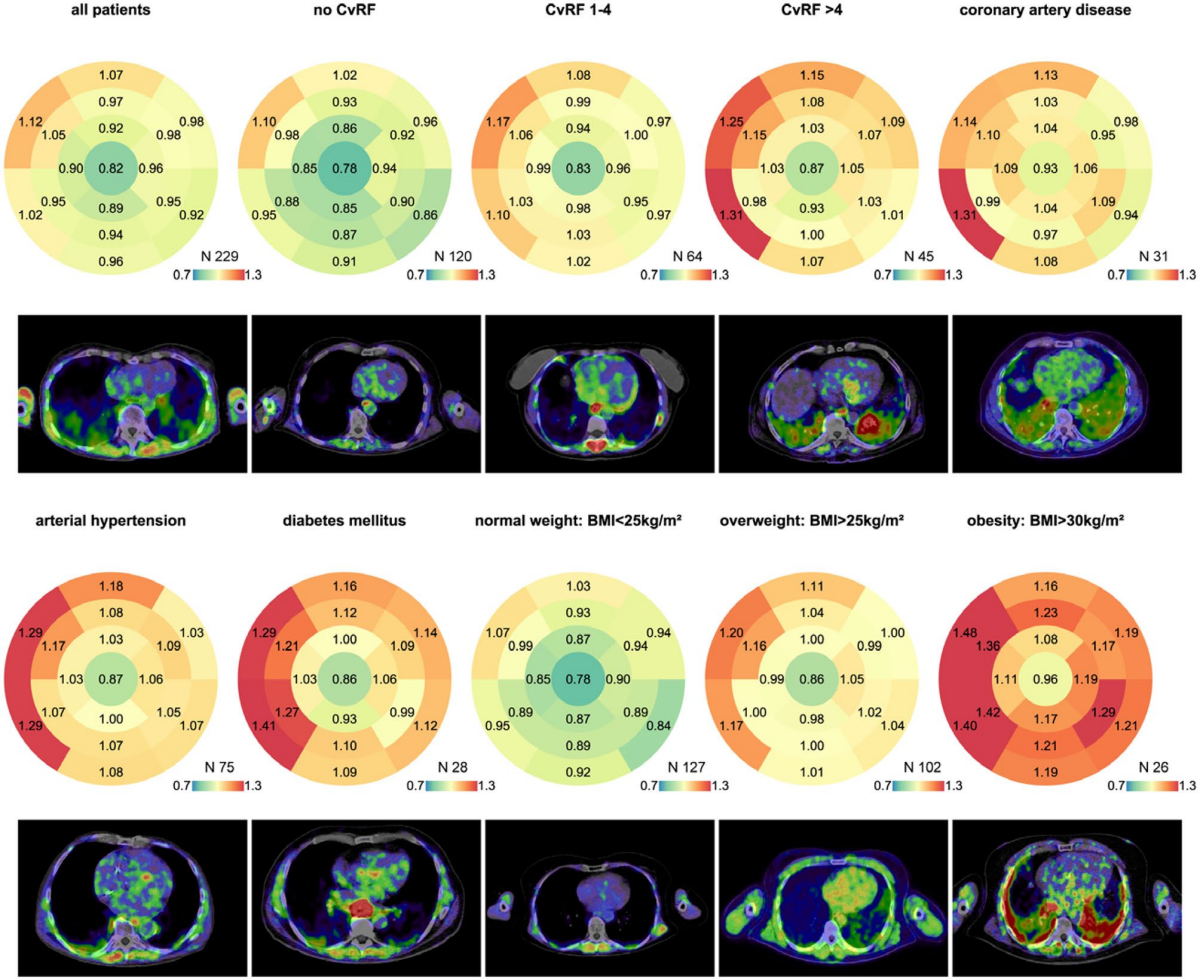
Retrospective assessment of oncology patients using 17 segment polar map analysis demonstrates progressive association of increasing cardiovascular risk factors (CvRF) with higher FAPI PET signal in the myocardium. The presence of coronary artery disease, arterial hypertension, or diabetes mellitus as well as rising body mass index (BMI) were further associated with higher FAPI signal particularly in the septal wall. Quantitative measurements are presented as median SUV, and a representative transaxial slice is presented below the polar maps. Reproduced from [26], with permission

Initial evaluation of the potential for FAP imaging in cardiovascular disease relied on selective analysis of images from oncology cohorts with concomitant cardiac injury. A plethora of case studies were reported over the last years describing remarkable uptake of FAPI ligands in the myocardium of cancer patients, related to cardiotoxicity, chemoradiotherapy, or hypertension [21–23]. Several retrospective studies have identified correlations between the cardiac FAPI standardized uptake value and the presence of coronary artery disease [24, 25]. Among 21 oncology patients, increased ^18^F-AlF-FAPI-04 in myocardium was associated with increased cardiac troponin I and worse ejection fraction [25]. In a study of 26 patients with immune checkpoint inhibitor therapy, 3 patients with suspected myocarditis exhibited higher FAPI signal in the myocardium relative to patients without evident cardiac disease. The cardiac signal was associated with elevated troponin T, abnormal electrocardiogram, and lymphocyte infiltration on biopsy [23]. In the largest such patient cohort, Heckmann and colleagues evaluated FAPI images from 229 oncology patients of mixed etiology subdivided to a modeling cohort and confirmatory cohort, exhibiting significant degrees of previously diagnosed coronary artery disease, hypertension, and type 2 diabetes mellitus [26]. Cardiac image enrichment patterns were described as homogeneous, diffuse, focal on diffuse, focal, or weak. Univariate regression modeling identified an association between focal left ventricle FAP and cardiac risk factors, presence of hypertension, known coronary artery disease, and medication with aspirin or statins (Fig. 2). Taken together, these clinical observations highlight a potential diverse role of FAP imaging to visualize progressive cardiac fibroblast activation and provide the foundation for dedicated cardiac imaging studies.

## Imaging FAP in Experimental Cardiovascular Disease

**Fig. 3:**
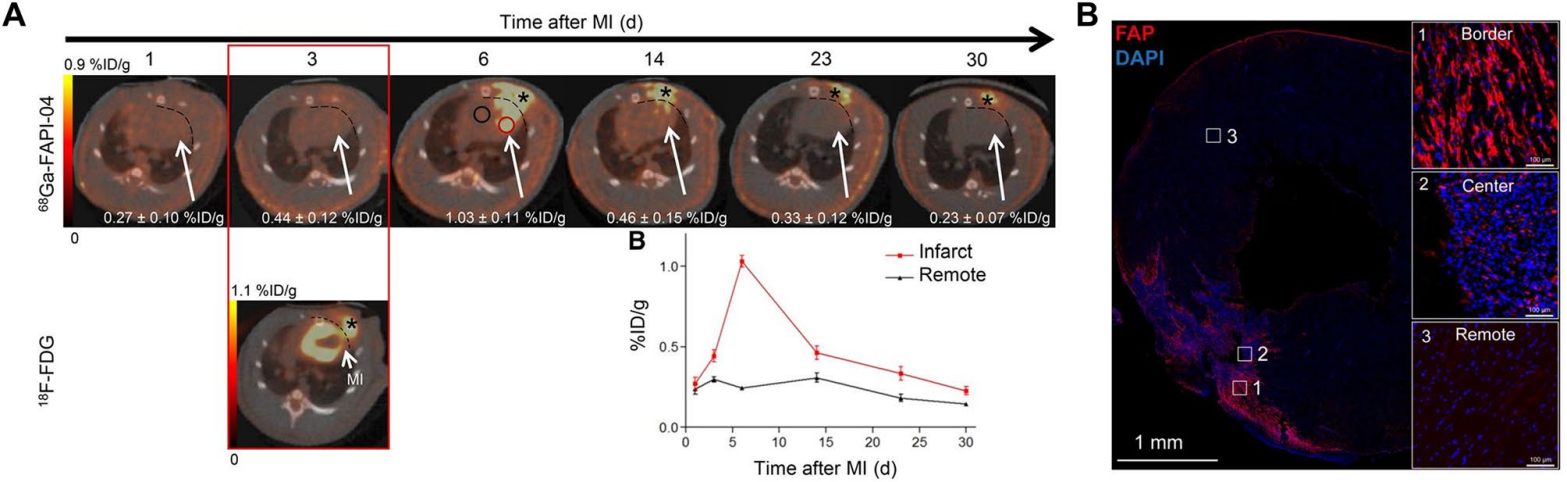
**A** Preclinical serial imaging of FAP upregulation using ^68^Ga-FAPI-04 in rats after coronary artery occlusion. Transient upregulation is identified in the infarct region (red circle) 6 days after myocardial infarction relative to remote non-infarcted myocardium (black circle). Persistent signal is observed at the site of the surgical wound (asterisk). **B** Fluorescence immunostaining localizes FAP expression to the infarct and border zone with limited staining in the non-infarcted myocardium. Reproduced from [27•], with permission

Preclinical imaging studies have enabled further characterization of FAP expression in the heart after injury. In a seminal study, Varasteh and colleagues performed longitudinal ^68^Ga-FAPI-04 imaging in rats after coronary artery occlusion over 4 weeks after injury. They reported specific FAPI signal in the infarct region which could be blocked by excess cold compound, reaching maximum at 6 days after myocardial infarction (Fig. 3). Immunohistochemistry identified localization of FAP in the infarct and border zone at 6 days after injury, colocalized to prolyl-4-hydroxylase β-positive proto-myofibroblasts moreso than to α-smooth muscle actin-positive myofibroblasts [27•]. High resolution autoradiography verified the strongest regional FAPI signal to derive from the infarct border zone [27•]. These observations have been corroborated by subsequent studies with increased frequency of FAPI imaging, with similar maximal signal at 6 days after coronary artery ligation in rats [28]. Likewise, temporal upregulation of FAP was identified in mice after myocardial infarction using an alternative FAP radiotracer ^68^Ga-MHLL1 [19]. Highest signal was observed in the infarct border zone at 7 days after myocardial infarction which remained elevated at 21 after injury compared to non-infarcted remote myocardium.

Experience outside of myocardial infarction is sparse, though a recent preclinical study evaluated FAPI signal in a rat model of pressure overload heart failure [29]. The authors described gradual increase in ^68^Ga-FAPI-04 signal in the global myocardium after abdominal aortic constriction which corresponded to declining cardiac function and altered ventricle geometry [29]. But minimal validation measurements limit the conclusions that can be drawn from this work.

Preclinical studies have thus provided some evidence of prognostic value of FAP imaging in cardiovascular disease, but the precise cellular substrate and temporal dynamics require further evaluation.

## First Clinical Experience of Cardiac FAP-Targeted Imaging

Direct experience of FAP imaging in cardiovascular patient populations is to date limited. Spurred in part by case study observations in myocardial infarction [30], preliminary evaluations in patients with primary cardiovascular disease have begun to emerge (Table 2).

**Table 2:** Cardiac applications with FAP agents

**Compound**	**Population**	**Observations**	**Ref**
Preclinical			
^68^Ga-FAPI-04	Myocardial infarction (rat)	Transient increase in infarct region signal, maximal at 6 d after occlusion	[27•]
	Isoproterenol (rat)	Transient increase in global cardiac signal after treatment, maximal at 7 d returning to baseline	[43]
	Abdominal aortic constriction (rat)	Persistent elevation in global cardiac signal at 2, 4, and 8 weeks of pressure overload	[29]
^68^Ga-MHLL1	Myocardial infarction (mouse)	Elevated uptake in the infarct region at 7 d and 21 d after occlusion, parallel elevation in remote non-infarcted myocardium	[19]
Clinical			
^68^Ga-FAPI-46	Myocardial infarction, < 11 d after infarct	Signal enrichment extending beyond perfusion defect, segments distinct from late gadolinium enhancement	[33••]
	Myocardial infarction, 4–16 d after infarct	Elevated signal in infarct region corresponding to affected vascular territory	[32]
^18^F-AlF-NOTA-FAPI-04	Myocardial infarction, 6 d after infarct	Higher tracer uptake in infarct region exceeding late gadolinium enhancement and T2 edema	[34]

**Fig. 4:**
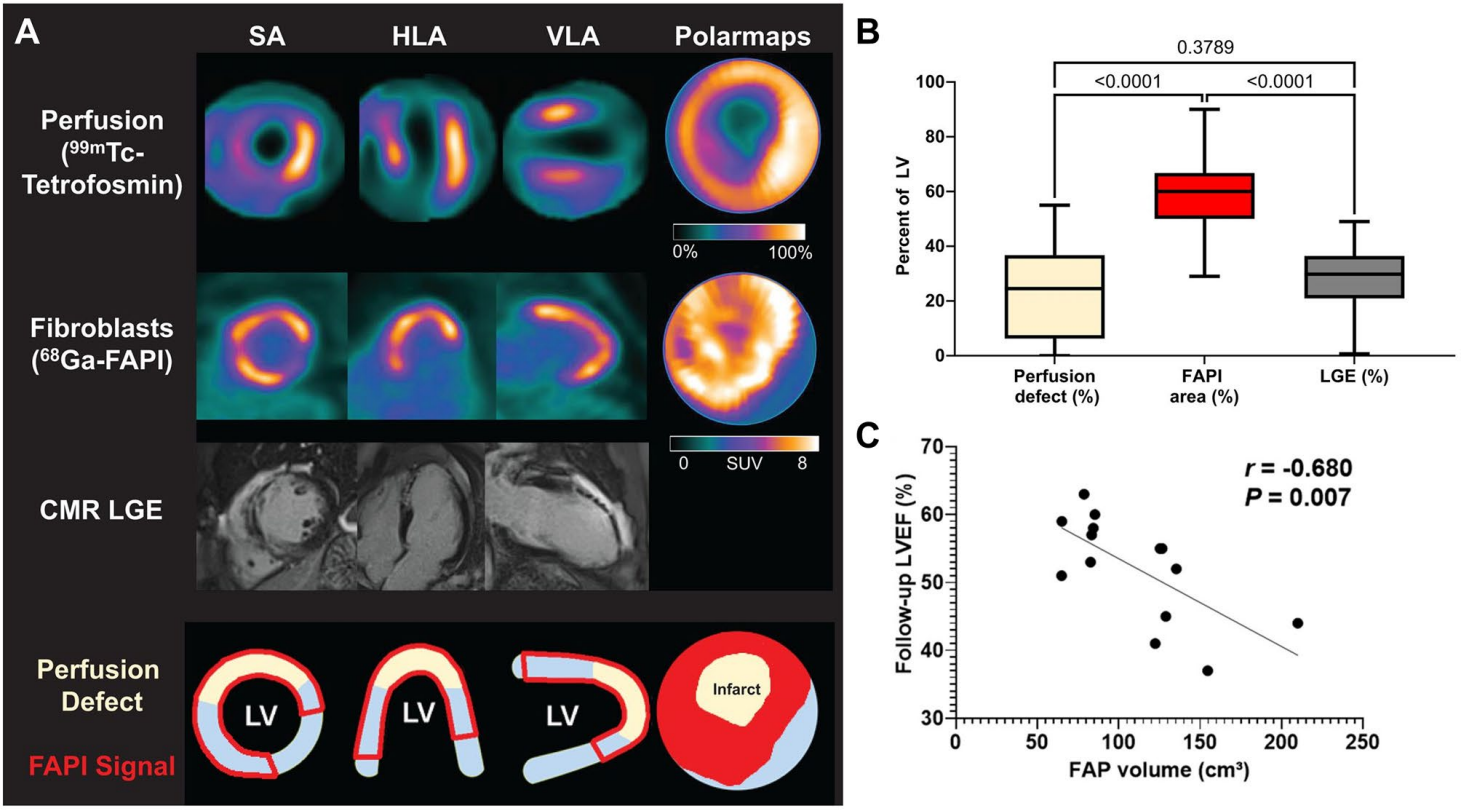
Clinical imaging with ^68^Ga-FAPI-46 after first myocardial infarction in patients. **A** Representative imaging workup and **B** quantitative analysis demonstrate that extent of FAPI upregulation exceeds the perfusion defect defined by myocardial perfusion imaging with tetrofosmin and scar defined by late gadolinium enhancement (LGE) on cardiac magnetic resonance (CMR). **C** The volume of FAP upregulation defined by PET imaging inversely correlates with subsequent left ventricle ejection fraction. Reproduced from [33••], with permission

An initial investigation of 12 patients after acute myocardial infarction identified extensive increased ^68^Ga-FAPI-46 signal in the infarct territory relative to blood and remote non-infarcted myocardium [31]. The extent of the FAP signal consistently exceeded the perfusion defect defined by ^99m^Tc-tetrofosmin single photon emission computed tomography (SPECT) and scar defined by late gadolinium enhancement on magnetic resonance imaging by an average of 30% of the left ventricle [31]. Similar visualization was reported in a different group of 10 acute myocardial infarction patients with and without ST segment elevation in which regional FAPI signal enrichment colocalized to the reperfused vessel on angiography [32]. The extent of the increased FAPI signal inversely correlated with contractile function at time of evaluation [32]. More recently, follow-up studies in 35 patients imaged within 11 days of acute myocardial infarction demonstrated that the regional FAP upregulation identified distinct myocardial segments from late gadolinium enhancement and prolonged T1 relaxation from matched cardiac magnetic resonance imaging [33••]. While FAPI-positive segments accounted for an average of 62% of the left ventricle, a significant portion of these segments exhibited normal T1 and T2 relaxation times indicating lack of tissue fibrosis and edema (Fig. 4). Accordingly, the FAPI signal defines a biologically distinct substrate, reflecting activated fibroblasts rather both in the infarct border zone and non-infarcted myocardium. Notably, the extent of FAP upregulation correlated with left ventricle ejection fraction 42–214 days after initial infarction [33••]. In a similar study, ^18^F-AlF-NOTA-FAPI uptake was observed in 14 patients 6 days after myocardial infarction compared to healthy controls. FAPI-positive area similarly exceeded late gadolinium enhancement and T2 signal in the left ventricle, and target-to-background FAPI signal correlated with magnetic resonance markers of edema scar and extracellular volume [34]. The maximum myocardial target-to-background ratio correlated with contractile function at 84-day follow-up.

In other cardiovascular disease, increased ^68^Ga-FAPI-04 uptake was identified in the right ventricle of patients with chronic thromboembolic pulmonary hypertension. Elevated target-to-background ratio was observed in the right ventricle free wall which corresponded to thickening of the affected wall but did not correlate with magnetic resonance imaging markers of established fibrosis [35]. Moreover, in the preliminary investigation in patients with high-grade aortic stenosis, the extent of an elevated FAP signal was greater compared to control patients and correlated with left ventricle ejection fraction and longitudinal strain [36]. As with myocardial infarction, FAP positive segments comprised regions with and without late gadolinium enhancement and prolonged T1 relaxation time. More recently, 80% of patients (*n* = 30) with light chain amyloidosis were found to exhibit increased ^68^Ga-FAPI-04 uptake in the left ventricle, in an extensive or patchy pattern. Increased uptake correlated with conventional biochemical markers of disease including Mayo Stage scoring, circulating BNP, echocardiography, and MRI parameters [37]. As such, diverse patient populations exhibit activated cardiac fibroblasts which may be amenable to monitoring by PET imaging and unlock new treatment options in these pathologies [38].

Taken together, these studies demonstrate that FAPI PET provides unique information on activated fibroblasts in the remodeling heart, with potential added value to predict outcome after, e.g., myocardial infarction. But these findings remain underdeveloped, wherein the appropriate timeframe for post-infarction imaging and quantification of images requires further investigation. The contribution of FAP signal in the infarct versus non-infarcted myocardium to subsequent remodeling and cellular substrates remains unclear. Moreover, clinical application in other cardiovascular conditions remains sparse, limited largely to case studies. As such, thorough and precisely designed prospective clinical imaging trials are necessary to fully understand the potential impact of this technique on patient management and treatment.

## Future Perspective and Conclusions

Importantly, modulation of fibroblast activation has been identified as a potential therapeutic avenue to curtail ventricle remodeling and improve outcomes. Indeed, FAP itself is a viable therapeutic target, wherein genetic deletion leads to less ventricle dilation and thicker scar formation [39]. Moreover, immunomodulatory therapy using chimeric antigen T cells to remove FAP-expressing cells in a mouse model of hypertension-induced cardiac fibrosis leads to a remarkable regression of interstitial collagen deposition [7•, 8]. The ability to monitor the response of FAP to targeted therapy is attractive, particularly considering the dual-edged nature of activated fibroblasts, which are both necessary for scar formation and wound healing but, when left unchecked, may become deleterious.

Realizing the full potential of FAP imaging for cardiovascular disease will require dedicated prospective clinical trials that establish the timecourse of FAP upregulation after initial injury and define the optimal timeframe for possible intervention. Improved quantification may enhance the power of imaging measurements, which have thus far relied on only extent of enhanced signal and crude target to background ratios that are poorly translatable across imaging centres. A further challenge to quantification is the relative lack of normal healthy control cardiac tracer uptake measurements. It is further paramount to delineate the cellular basis of the FAP imaging signal, to demarcate the fibroblast subpopulations that are potentially beneficial from those that are detrimental. Integration of imaging with spatial omics technologies can provide valuable insights into the molecular basis of the imaging signal, which can thereby stimulate novel therapeutic strategies. Furthermore, to establish clinical utility, the tracer signal must also respond to therapeutic intervention targeted at FAP or other fibrosis mechanisms. Pre- and post-therapy measurements in animal models of disease or patient cohorts will provide greater impetus for implementing FAP imaging into clinical practice.

The feasibility of visualizing activated cardiac fibroblasts using FAP-targeted radioligands has been now clearly demonstrated. The challenge is to transform this potential into clinical reality, through dedicated and thorough pre-clinical and clinical investigation beyond retrospective analyses and case reports. With an established foundation, it is time to build upward to realize the potential of FAP-targeted imaging in cardiac applications.
